# GC-MS analysis of *Myrtus communis* extract and its antibacterial activity against Gram-positive bacteria

**DOI:** 10.1186/s12906-020-2863-3

**Published:** 2020-03-17

**Authors:** Mushtaq A. Mir, Nasreena Bashir, Abdulkhaleg Alfaify, Mohammed D. Y. Oteef

**Affiliations:** 1 Department of Clinical Laboratory Sciences, College of Applied Medical Sciences, King Khalid University, P. O. Box 3665, Abha, 61421 Saudi Arabia; 2 Department of Biology, College of Science, King Khalid University, P. O. Box 9004, Abha, 61421 Saudi Arabia; 3 Department of Chemistry, College of Science, Jazan University, Jazan, 82817 Saudi Arabia

**Keywords:** Plant extract, *Myrtus communis*, Gram-positive bacteria, Gram-negative bacteria, GC-MS, Cell wall

## Abstract

**Background:**

*Myrtus communis* is a typical plant of Mediterranean area. The different parts of this plant such as berries, branches, and leaves have been used worldwide as a traditional/folk medicine for the treatment of various ailments and diseases.

**Methods:**

Ethanolic leaf extract of the plant was prepared by Soxhlet extraction method. Zone of inhibition, minimum inhibitory concentration and minimal bactericidal concentration were determined by well diffusion method and microplate alamar blue assay. GC-MS analysis was carried out to identify the compounds present in the extract. Microscopy and ImageJ software were used respectively for morphology and cell-length measurements. GraphPad Prism was used for statistical analysis.

**Results:**

The ethanolic extract showed strong inhibitory effect against Gram-positive and acid-fast bacteria with significant inhibition-zone size (9–25 mm), MIC (4.87–78 μg/ml), as well as MBC (0.3–20 mg/ml). However, no effect was observed on the growth of Gram-negative bacteria. The growth inhibition was found to be associated with the damage of cell wall as the extract-treated cells were sensitive to cell wall-targeting antibiotics and displayed the cell wall damage-depicting morphological defects. GC-MS analysis confirmed the presence of novel compounds in addition to the most representative compounds of the essential oils/extracts of *M. communis* of other country origins.

**Conclusion:**

These results demonstrate that *M. communis* leaf extract could be the source of compounds to be used for the treatment of Gram-positive bacterial infections. This is the first report, which provides insights into the mechanism of action of the extract in inhibiting the growth of Gram-positive bacteria.

## Background

Plant extracts and plant-derived products are valuable sources that are widely used to treat a wide range of disease conditions caused by bacterial and fungal pathogens [1, 2]. *Myrtus communis L*. (Arabic name: Aas or Hadas) is a typical evergreen leafy perennial sweet-smelling shrub or small tree belonging to the family Myrtaceae. It is native to the Mediterranean region along with other countries including the Middle East countries such as Jordan, Iraq, and Saudi Arabia [3]. Different parts of this plant such as its berries, branches, and leaves have been used worldwide as a traditional/folk medicine for the treatment of various ailments and diseases [4]. Essential oils and extracts obtained from different parts, *viz.,* berries and leaves of *M. communis* from different countries of its origin inhibited the growth of several pathogenic bacteria as beautifully summarized in a review article by Aleksic, V and Knezevic, P [3]. Besides its use in traditional medicine, *M. communis* has been extensively used in perfumery, foods, cosmetics, spices, and pharmaceutical industries [5]. Various myrtle extracts, fractions, and phyto constituents are known to be used in several remedies like anticancer [6], antibacterial [3], antioxidants [7], and analgesic [8]. The leaves of the plant are used as anti-inflammatory and antiseptic agent, as well as in the treatment of urinary, oral and respiratory diseases [9, 10]. It has been reported that different parts of the plant are rich sources of various bioactives [11–13]. Chemical constituents of *M. communis* from different parts of the world are reported to differ significantly [14]. In Saudi Arabia, *M. communis*, the local name ‘Musk Al-Medina’ has not been studied in detail, though used widely in perfumery industries as well as in traditional medicine for the treatment of jaundice, liver disorders, nausea, lack of appetite and other stomach illnesses [15]. In this study, we investigated its antibacterial activity against wide range of bacterial strains including Gram positive, Gram negative, and actinobacteria. Gram-positives were found to be highly sensitive, while Gram negatives were found to be resistant to the bioactives of the ethanolic extract of *M. communis* leaves. Further investigation of the antibacterial effect of the extract led insights into the mechanism of action of its constituents targeting the cell wall as is evident by inhibition of the bunching phenotype of *S. aureus* and shortening of the cell length of *M. smegmatis*, both of which are related to cell wall biogenesis. GC-MS analysis of the extract detected 50 compounds, most of which are found in same plant type of other country’s origin. Several of these compounds are documented to have antibacterial activity. Resistance acquired by human pathogens to the presently available commercial drugs is a major health issue. One of the possible ways to treat these antibiotic resistant bacterial infections is to identify the novel compounds of the medicinal plants used in traditional medicine. Isolation of the compounds from *M. communis* and determination of their antibacterial activity individually or in combination with already available drugs might help in treating the drug resistant human pathogens by any of the possible way(s) as discussed in the review by Ayaz et al. [16].

## Methods

### Preparation of plant extract

The dry leaves of the medicinal plant *M. communis* native of Faifa Mountain located in the east of Jazan, Saudi Arabia, were grinded into a fine powder. No permission was required to obtain the aerial parts of the plant for its identification and extract preparation. The specimen was identified and confirmed by a taxonomist, Dr. Boulbaba L’taief, Biology Department, College of Science, King Khalid University, Abha, Saudi Arabia, for authenticity. The voucher sample was submitted to the herbarium of the Department of Biology at King Khalid University Abha, Saudi Arabia to obtain the voucher number (#45657). The extract was prepared in ethanol by Soxhlet extraction method. In brief, 50 g of the leaf powder were incubated with 200 ml of absolute ethanol for 2 hours in Soxhlet extractor. The extract was filtered through Whatman-1 paper and the filtrate thus obtained was poured in petri dishes. The petri dishes were left open at room temperature till complete evaporation of ethanol. The dried extract of *M. communis* was re-dissolved in ethanol at 0.4 g/ml, which was further diluted by 2-fold dilutions to obtain stock solutions ranging from 0.4 g/ml to 3 μg/ml. These stock solutions were used later for MIC determination. The same extract dissolved in ethanol was used for GC-MS analysis.

### Microorganisms and media

Ten bacterial strains including laboratory and reference strains, *viz*., *Staphylococcus aureus*, *Staphylococcus epidermidis* (ATCC12228), *Pseudomonas aeruginosa* (ATCC9027), *Escherichia coli* (ATCC25922), *Enterococcus faecalis*, *Enterococcus faecalis* (ATCC29212), *Klebsiella pneumoniae*, *Salmonella typhi, Shigella* and *Mycobacterium smegmatis* mc^2^155, were used in this study. Fungal strain *Candida albicans* was also used. The strains included both Gram-positive and Gram-negative strains, as well as an acid-fast strain *M. smegmatis* mc^2^155. All strains were grown in nutrient broth media, except *M. smegmatis*, which was grown in Middlebrook 7H9 broth. The MIC for all strains was determined by the broth dilution method using alamar blue as a growth indicator.

### Zone of inhibition by well diffusion method

For the initial screening of bacterial strains for their sensitivity to extract, the strains were grown to the logarithmic phase of growth (O.D_600_ 0.4–0.6) in nutrient broth. Subsequently, the strains were diluted in nutrient broth to a theoretical OD_600_ of 0.01. Well diffusion method [17] was used to determine the zone of inhibition. In brief, the diluted culture was uniformly spread on nutrient agar plates using a sterile cotton swab. Wells (diameter 6 mm) were made in agar by the sterile syringe-needle cap. 20 μl of either extract (0.4 g/ml) or ethanol were dispensed in triplicate wells. Plates were incubated at 37 °C for 24 hours. Diameter of the clear zone of inhibition, which includes the well diameter, was measured. Actual zone of inhibition was calculated by subtracting the average zone of inhibition by ethanol from average zone of inhibition by the extract.

### Microplate alamar blue assay (MABA)

To determine the antimicrobial activity of plant extract, the minimum inhibitory concentration (MIC) was determined by MABA [18]. In brief, the bacterial cultures grown to the logarithmic phase of growth (O.D_600_ 0.4–0.6) were diluted in nutrient broth to a theoretical O.D_600_ of 0.01. Subsequently, 190 μl culture of each bacterial strain was dispensed in the wells of 96-well plate. 10 μl from the 2-fold diluted plant extract stocks ranging from 0.4 g/ml to 3 μg/ml were added in triplicate wells. For vehicle control, 10 μl of ethanol was added in triplicate wells. 5% of ethanol was found to have a minimal detrimental effect on the growth of bacteria especially *M. smegmatis*. To avoid evaporation, the peripheral wells were filled with water and a tray of water was put inside the incubator. The plates were incubated at 37 °C in the incubator for 24 hours. After 24 hours of incubation, 20 μl of sterile alamar blue (Thermo Fisher Scientific) was added to each well and the development of pink color was monitored after every 1 h. The minimal concentration of the plant extract in a well, which did not change color from blue to pink, was considered as minimum inhibitory concentration. To determine the minimum bactericidal concentration (MBC) of the extract for a bacterial strain, 10 μl from the culture wells wherein the blue color of Alamar blue remains unchanged and of the control wells (pink color) were streaked on nutrient agar plates. The concentration of the extract where no growth was detected was considered as MBC of the extract for the strain tested.

To determine the effect of various antibiotics on the extract-treated bacterial cells, 1 ml of the bacterial culture (OD_600_ of 0.2) was treated with the 4xMIC of extract for 45 min at 37 °C. The cells were washed twice with PBS and resuspended into the 1 ml of nutrient broth. The culture was diluted to the theoretical OD_600_ of 0.01 in the same culture media containing Alamar blue at 1/10^th^ dilution. The 190 μl of the culture was dispensed into the wells of 96 well plates. The antibiotics tetracycline, levofloxacin, and colistin were added individually in triplicate wells each at a concentration of 1/8^th^ of their corresponding MICs. Absorbance was measured at 570 nm in 96 well plate reader after either every 30 min (*S. aureus*) or 3 hours (*M. smegmatis*). The culture wells without any antibiotic were taken as control. The average of the absorbance was plotted against time.

### GC-MS analysis

Gas chromatography-mass spectrometry (GC-MS) analysis was carried out using a GC-MS apparatus (model; QP2010 Ultra, Shimadzu Corporation, Kyoto, Japan). The sample components were separated on a 30 m length × 0.25 mm i.d. capillary column coated with a 0.25 μm film thickness stationary phase (Rtx-5MS, Restek Corporation, Bellefonte PA, U.S.A). Helium (99.999%) was employed as carrier gas at a constant linear velocity of 36.3 cm/sec. The sample volume of 1 μl was injected using AOC-20i + s autoinjector. The injection port was set at 290 °C in splitless mode. The GC oven temperature was programmed as follows: 5 min. at 50 °C, heated at 2 °C/min. to 300 °C and held for 10 min.

The ion source temperature in the MS was set at 230 °C and the Interface at 280 °C. Total Ion Chromatogram (TIC) was created for m/z range 30 - 700. GC peaks were identified by comparing their mass spectra to the database of the National Institute of Standards and Technology (NIST). The relative percentage amount of each component was calculated by comparing its peak area to the total area of peaks in the chromatogram.

### Microscopy

For microscopy, cells after treatment with ethanol or the extract were washed once with the growth media and immediately observed under the 100x objective of Nikon microscope, fitted with 10MP USB 2.0 color CMOS C-mount camera. DIC images were taken for both control and treated cells. The length of more than 100 cells of *M. smegmatis* for each condition (ethanol and extract-treated) was measured by ImageJ software. For statistical analysis unpaired t-test with 95% confidence interval of − 1.32 to - 0.88 using GraphPad Prism, version 8.1.2 was used.

## Results

### Strong antibacterial activity of the extract against Gram-positives

In order to investigate the antibacterial activity of the *M. communis* extract, both the laboratory and reference strains including Gram-positive, Gram-negative and acid-fast were treated with two-fold dilutions of extract ranging from 0.4 g/ml to 3 μg/ml. After 24 hours of incubation, the growth was detected by adding alamar blue at 1/10^th^ dilution. The change in color from blue to pink indicates the active growth of cells. The minimum concentration of the extract at which the blue color of the dye remained unchanged was considered as the minimum inhibitory concentration. It is quite evident from Table 1 that the Gram-positives were highly sensitive to the secondary metabolites of the leaf extract, while as Gram-negatives were resistant. The growth of Gram-positive bacteria was inhibited with MIC ranging from 4.87 to 78 μg/ml and MBC ranging from 0.3 to 20 mg/ml. These results corroborate very well with corresponding higher zones of inhibition ranging from 9 to 25 mm (Table 1). Interestingly, the MIC of the *M. communis* leaf extract against the strains tested in this study is very low than that of leaf essential oil or extract of the same plant from the other Mediterranean region countries [19–21]. These results confirm that the Gram-positive strains were sensitive, while Gram-negative strains were resistant to the extract.

**Table 1:** Effect of ethanolic leaf extract of *M. communis* on different bacterial and fungal strains

Organisms	Zone of inhibition (mm) ± SD	MBC (mg/ml)	MIC (μg/ml)
Gram positives			
*Enterococcus faecalis* (*ATCC29212*)	9 ± 1.2	5	78
*Enterococcus faecalis*	9 ± 1.2	5	19.5
*Staphylococcus aureus*	25	0.3	9.7
*Staphylococcus epidermidis* (*ATCC12228*)	15 ± 1	20	4.87
*Mycobacterium smegmatis mc*^*2*^*155*	17.6 ± 0.7	1.2	19.5
Gram negatives			
*Pseudomonas aeruginosa* (*ATCC9027*)	–	–	–
*Salmonella typhi*	–	–	–
*Escherichia coli* (*ATCC25922*)	–	–	–
*Shigella*	–	–	–
*Klebsiella pneumonia*	–	–	–
Fungi			
*Candida albicans*	–	–	–

(−); no inhibition

### Ethanolic leaf extract targets the cell wall

**Fig. 1:**
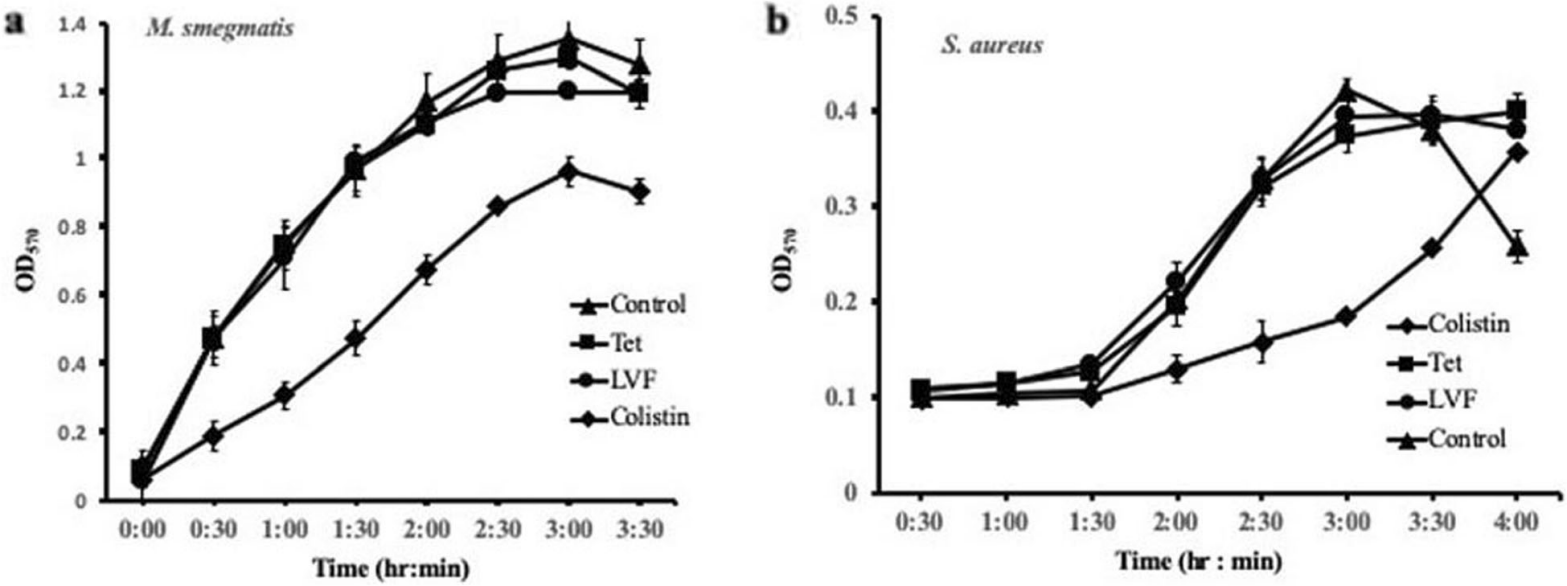
Sensitivity of extract-treated *M. smegmatis* and *S. aureus* cells to antibiotics. Cells after treatment with ethanolic extract were washed and re-suspended in nutrient broth containing 1x alamar blue. The cultures were aliquoted in triplicate wells of a microplate containing an antibiotic (colistin, tetracycline or levofloxacin). For the control, no antibiotic but water was added. Absorbance at 570 nm was measured at regular time intervals of 3 hours (*M. smegmatis*) or 30 min (*S. aureus*). For each antibiotic, the average values of A_570_ (YY-axis) of the extract-treated *M. smegmatis* (**a**) or *S. aureus* (**b**) culture were plotted over the time (XX-axis). Error bars; ± SD

Growth inhibition of especially Gram-positive bacterial strains by the extract prompted us to look at whether or not the inhibition was due to the effect on cell wall biogenesis. We performed the real-time investigation of the sensitivity of the extract-treated bacterial cells to the antibiotics targeting cell wall synthesis, protein synthesis, and DNA replication/supercoiling. Extract-treated cells of *S. aureus* and *M. smegmatis* were washed and incubated individually with different types of antibiotics, *viz.*, colistin (cell wall synthesis inhibitor), tetracycline (protein synthesis inhibitor), and levofloxacin (DNA replication/supercoiling inhibitor) at the concentration of 1/8^th^ of their respective MICs. The growth of *S. aureus* was monitored at time intervals of 30 min (Fig. 1a), while that of *M. smegmatis* was monitored at regular intervals of 3 hours (Fig. 1b). It is quite evident that the growth of both the bacterial strains was sluggish to the treatment of colistin, while as other antibiotics on either of the two strains observed no effect, suggesting, the crude extract targeted the cell wall.

Furthermore, *M. smegmatis* and *S. aureus* bacterial strains were individually treated with 2-fold dilutions of above-mentioned antibiotics alone and in combination with 1/16^th^ MIC concentration of the plant extract. It was found that in the presence of extract the MIC of only colistin against *M. smegmatis* and *S. aureus* was decreased 64-fold and 32-fold, respectively, while as no effect was observed on the MIC of other two antibiotics (tetracycline and levofloxacin) against either of the bacterial strains (Table 2). Similar to colistin, the leaf extract enhanced the effect of other cell wall targeting antibiotic vancomycin, although the enhancing effect was moderate. In the presence of leaf extract, the MIC of the vancomycin for *M. smegmatis* and *S. aureus* decreased to 1/8^th^ and 1/4^th^ of its MIC in absence of leaf extract (Table 2). These results support the fact that the plant extract targeted the cell wall.

**Table 2:** MICs of various antibiotics alone and in combination with the ethanolic leaf extract of *M. communis* (conc.; 1/16^th^ of its MIC) against *M. smegmatis* and *S. aureus*

Antibiotic	*M. smegmatis*		*S. aureus*	
	MIC (μg/ml)	MIC (μg/ml) in presence of extract	MIC (μg/ml)	MIC (μg/ml) in presence of extract
Colistin	1.25	0.0195	1.56	0.048
Vancomycin	0.37	0.047	12.5	3.13
Tetracycline	0.039	0.039	1.56	1.56
Levofloxacin	0.078	0.078	3.12	3.12

### *M. communis* leaf extract affected the cellular morphology

**Fig. 2:**
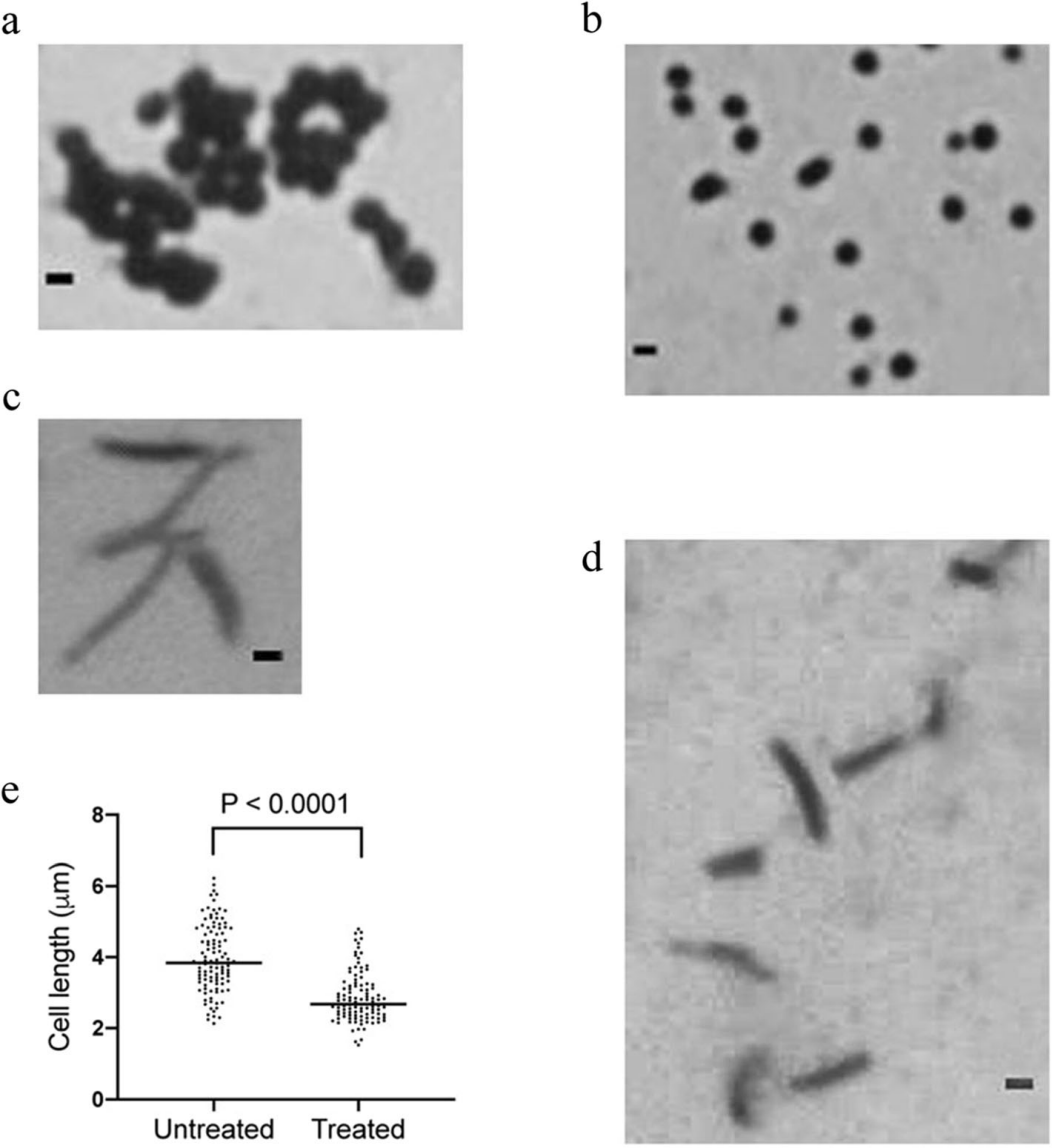
Morphology of the extract-treated *S. aureus* and *M. smegmatis* cells. Cells of *S. aureus* and *M. smegmatis* were individually treated with ethanolic leaf extract of *M. communis*. For the control, cells were treated with ethanol only. Both the control and extract treated cells were observed under the microscope using the 100x objective. (**a** & **b**) *S. aureus* cells treated with ethanol and extract, respectively. (**c** & **d**) *M. smegmatis* cells treated with ethanol and extract, respectively. (**e**) Distribution of *M. smegmatis* cells based on their length after treating separately with ethanol (untreated) and leaf extract (treated). Scale bar; 1 μm

To investigate the effect of the extract on the cellular morphology, the *S. aureus* and *M. smegmatis* cells treated with the extract at its MBC for 6 hours were observed under the microscope. It is evident from the Fig. 2 that compared to the bunching phenotype of the control cells of *S. aureus* the treated cells appeared as individual cells. While as for *M. smegmatis*, the treated cells remarkably appeared shorter than the control cells and the data was statistically significant (*p* < 0.0001). These results concluded that due to the cell wall defect the morphological properties of cells are affected, as cell wall maintains the cell’s physical properties of shape and size.

### Chemical composition of the ethanolic leaf extract of *M. communis*

The compounds identified in the leaf extract by GC-MS analysis are listed in the order of their column elution time (Table 3). In *M. communis* leaf extract 50 compounds were detected, which constitute 71% of the whole extract. The most dominant of all the identified compounds were 1,1,8a-trimethyloctahydro-2,6-naphthalenedione (27.6%), pyrogallol (9.1%), 1,8-cineole (3.9%), while the most representative compounds identified were α-terpineol (1.6%), linalool (2.8%), squalene (1.15%), α-terpinyl acetate (1.02%), D-limonene (0.65%) and linalyl acetate (0.97%). The other constituents present in appreciable amounts were β-caryophyllene (0.56%), acetol (0.64%), linalyl formate (1.93%) and α-tocopherol-β-D-mannoside (1.78%).

**Table 3:** Relative percentage of the compounds detected in ethanolic leaf extract of *M. communis* by GC-MS

S. No.	Rt^*^ (min)	Compound Name	SI^**^	% Peak area^***^
1	5.1	Acetol	98	0.64
2	6.2	Methyl acrylate	91	0.50
3	6.5	Methyl acetate	91	0.19
4	6.7	Ethyl glycolate	96	0.13
5	6.9	Methyl pyruvate	93	0.57
6	7.3	Ethyl orthoformate	85	1.99
7	8.7	3-Hydroxymethylfuran	94	0.17
8	9.5	Isopropyl isopropoxyacetate	80	0.36
9	9.9	Dihydroxyacetone	93	1.01
10	10.5	Ethyl diethoxyacetate	82	0.23
11	11.4	1,2-Cyclopentanedione	97	0.32
12	13.3	5-MethyIfurfural	96	0.10
13	13.8	(−)-β-Pinene	95	0.07
14	14.0	2,4-Dihydroxy-2,5-dimethyl-3(2H)-furanone	81	0.25
15	14.1	5-Diethoxymethyl-3-ethoxy-4,5-dihydro-isoxazole	83	0.12
16	14.4	Phenol	90	0.04
17	14.6	5-Diethoxymethyl-3-ethoxy-4,5-dihydro-isoxazole	80	0.14
18	14.8	Glutaconic anhydride	91	0.09
19	15.1	2,2-Diethyl-3-methyl-1,3-oxazolidine	80	0.06
20	16.7	D-Limonene	93	0.65
21	16.9	1, 8-Cineole	95	3.96
22	20.3	5-Hydroxyazouracil	84	0.17
23	20.4	(+)-4-Carene	90	0.18
24	21.3	Linalool	97	2.80
25	24.2	4H-Pyran-4-one, 2,3-dihydro-3,5-dihydroxy-6-methyl-	90	0.49
26	27.1	α-Terpineol	98	1.60
27	27.4	L-α-Terpineol	96	1.12
28	28.5	Catechol	96	0.49
29	30.5	5-Hydroxymethylfurfural	93	1.62
30	31.6	Linalyl formate	93	1.93
31	31.9	Linalyl acetate	91	0.97
32	38.0	α-Terpinyl acetate	92	1.02
33	40.4	Pyrogallol	92	9.11
34	41.7	Methyleugenol	92	0.12
35	42.2	β-Caryophyllene	94	0.56
36	42.7	α -Isomethyl ionone	82	0.21
37	43.5	Tyrosol	94	0.11
38	48.0	Cyclohexanecarboxaldehyde, 6-methyl-3-(1-methylethyl)-2-oxo-1-(3-oxobutyl)-	81	0.25
39	50.5	1,1,8a-Trimethyloctahydro-2,6-naphthalenedione	67	27.60
40	64.3	3-Methyl-2-butenoic acid, undec-2-enyl ester	82	0.81
41	65.8	Phytol acetate	91	0.42
42	71.7	Aspidinol	91	0.08
43	72.0	L-Ascorbyl 2,6-Dipalmitate	91	0.66
44	78.8	Phytol	85	0.19
45	79.9	9-Octadecenoic acid	88	0.30
46	94.9	Palmitic acid- β-monoglyceride	87	0.22
47	106.3	Squalene	94	1.15
48	115.1	n-Dotriacontane	95	0.72
49	116.1	α-tocopherol-β-D-mannoside	96	1.78
50	121.4	Clionasterol	84	1.35

*Rt: Retention time

**SI (Similarity Index): It is the percentage of computer-based spectral matching between the mass spectrum of each unknown peak in the chromatogram to the spectra stored in NIST library database. SI = 100; when two spectra are identical and 0 if they are completely different

***% Peak Area: It is the relative percentage amount of each component in the extract calculated by comparing its peak area to the total peak areas in the chromatogram

The identity of the most abundant compound 39 needs to be confirmed by further isolation and fractionation

The presence of newly identified compounds especially 1,1,8a-trimethyloctahydro-2,6-naphthalenedione (27.6%) and pyrogallol (9.1%) or their combination with other secondary metabolites might contribute to the specific killing of Gram-positives.

## Discussion

In this study, we investigated the antibacterial activity of the ethanolic leaf extract of *M. communis* of Jazan origin, the southwestern region of Saudi Arabia. The extract inhibited the growth of several bacterial strains of Gram-positive and acid-fast *M. smegmatis*. More interestingly, the antibacterial activity exhibited was stronger (in μg/ml level) compared to the extracts/essential oils obtained from the *M. communis* plants from other Mediterranean countries. The MIC of *M. communis* leaf essential oil of Ethiopian, Algerian and NW Morocco origin in mg/ml range is in support of this observation [19, 22, 23]. More interestingly the extract being bactericidal against Gram-positive bacteria was ineffective against Gram-negative bacteria and the fungal strain *Candida albicans.* Contrarily, Mediterranean country origin plants do exhibit antibacterial activity against Gram-negatives albeit lower than Gram-positives. Strong inhibition of growth of *M. smegmatis* is in support with the earlier observation wherein growth inhibition of the same genus species *M. tuberculosis* and *M. avium* was observed by essential oil of *M. communis* leaves [24].

It is often reported that the less sensitivity of Gram-negative to the extract and essential oils of plants is probably due to the presence of outer hydrophilic lipo-polysaccharide (LPS) layer that blocks the penetration of hydrophobic essential oils and avoids its accumulation in the target cell membrane [25, 26]. However, in the present study the bactericidal effect was found to be attributed to the damage of cell wall of Gram positive bacteria which do not possess the outer hydrophilic LPS, suggesting, that the target of the secondary metabolites of ethanolic extract of *M. communis* leaves might be the peptidoglycan layer rather than the penetration and accumulation of compounds in the target cell membrane. This is supported by the fact that Gram positive and acid fast bacteria, which are rich in peptidoglycan in their cell wall, were sensitive to the cell wall-targeting antibiotic rather than the antibiotics that target other vital and fundamental process of the cell *viz*., transcription/replication and protein synthesis.

The sensitivity of Gram-positives and acid-fast bacteria to the leaf extract in the present study could be attributed to the presence of new compounds detected by GC-MS analysis. However, variation in methods of extract preparation cannot be ruled out. Beyond the detection of the most representative compounds, the newly identified compounds such as 1,1,8a-trimethyloctahydro-2,6-naphthalenedione (27.6%) and pyrogallol (9.1%) were abundant. Their presence in leaves of *M. communis* of Saudi origin might be due to varying environmental factors such as geography, temperature, day length, nutrients, rainfall, type of soil as well as genetic dynamics of the inhabitant plants. The new compounds identified in the leaves of *M.communis* in this study would be ideal to try for antibacterial activity, as there are reports wherein purified compounds with the skeleton backbone of naphthalenedione or pyrogallol have been shown to inhibit the growth of bacteria [27–34]. Several studies have documented antibacterial activity of the pyrogallol and naphthalenedione derivatives. In one such study, pyrogallol was found to be second after curcumin among 48 polyphenols to inhibit both the planktonic and biofilm form of growth of periodontopathic bacteria [34]. In other study inclusion of pyrogallol in antibacterial films prepared from matrix of sodium alginate and carboxymethyl cellulose was found to be more effective against *E. coli* and *S. aureus* [29]. Furthermore, pyrogallol-rich polyphenols among about two dozens each of pure polyphenols and polyphenol-rich plant extracts showed a strong antibacterial activity against various bacterial strains tested [28]. Naphthalenedione derivative 5-hydroxy-1,4-naphthalenedione commonly known as juglone of teak bark inhibited several Gram positive bacteria notably *L. monocytogenes* and MRSA [32]. Similarly, in another study, 1,4-naphthalenedione isolated from leaf extract of a medicinal plant *Holoptelea integrifolia* was found to be effective against β*-*lactam resistant strain of *S. aureus* [31]. These reports are in well agreement with the antibacterial activity of the bioactives of *M. communis* extract presented in this study. However, it demands further isolation and fractionation to purify these compounds in order to determine their antibacterial activity individually and/or in combination.

## Conclusion

Few interesting and new findings of this study are 1) Though different parts of the *Myrtus communis* have been used worldwide as a traditional/folk medicine for the treatment of various diseases, very strong antibacterial activity of the ethanolic leaf extract of this plant in the present study was observed against Gram-positive and acid-fast bacteria as is evident by comparatively higher inhibition-zone sizes, and lower MICs and MBCs. 2) Interestingly, Gram-negative bacteria were resistant to the same extract. 3) The growth inhibition was associated with the damage of cell wall because of the sensitivity of extract-treated cells to the cell wall targeting antibiotic and displaying of the morphological defects. 4) GC-MS analysis confirmed the presence of few novel compounds in the extract. 5) This is the first report, which provides insights into the mechanism of action of the extract in inhibiting the bacterial growth. These results demonstrate that the *M. communis* leaf extract could be the source of compounds to be used for the treatment of Gram-positive bacterial infections.

## Data Availability

All data generated or analysed during this study are included in this article.
